# Encapsulation of Pt(iv) prodrugs within a Pt(ii) cage for drug delivery†
†Electronic supplementary information (ESI) available: Experimental details of synthesis and characterization of **1–3**, cell culture, the MTT assay, flow cytometric analysis, and fluorescence microscopic characterization. See DOI: 10.1039/c4sc01892c



**DOI:** 10.1039/c4sc01892c

**Published:** 2014-11-24

**Authors:** Yao-Rong Zheng, Kogularamanan Suntharalingam, Timothy C. Johnstone, Stephen J. Lippard

**Affiliations:** a Department of Chemistry , Massachusetts Institute of Technology , Cambridge , Massachusetts 02139 , United States . Email: lippard@mit.edu

## Abstract

This report presents a novel strategy that facilitates delivery of multiple, specific payloads of Pt(iv) prodrugs using a well-defined supramolecular system. This delivery system comprises a hexanuclear Pt(ii) cage that can host four Pt(iv) prodrug guest molecules. Relying on host–guest interactions between adamantyl units tethered to the Pt(iv) molecules and the cage, four prodrugs could be encapsulated within one cage. This host–guest complex, exhibiting a diameter of about 3 nm, has been characterized by detailed NMR spectroscopic measurements. Owing to the high positive charge, this nanostructure exhibits high cellular uptake. Upon entering cells and reacting with biological reductants such as ascorbic acid, the host–guest complex releases cisplatin, which leads to cell cycle arrest and apoptosis. The fully assembled complex displays cytotoxicity comparable to that of cisplatin against a panel of human cancer cell lines, whereas the cage or the Pt(iv) guest alone exhibit lower cytotoxicity. These findings indicate the potential of utilising well-defined supramolecular constructs for the delivery of prodrug molecules.

## Abstract 2

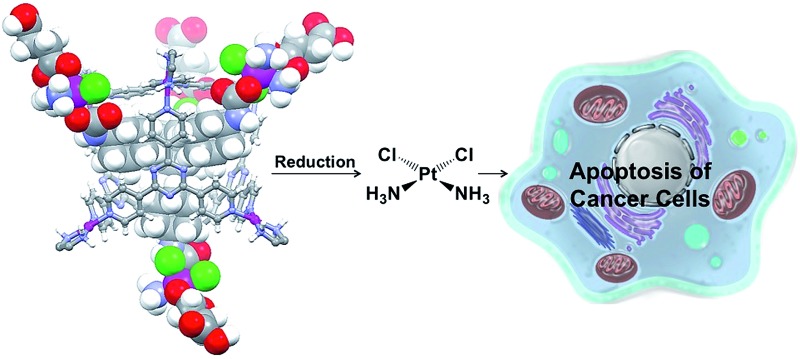
This report describes a novel strategy for delivery of adamantyl-functionalized payloads using a supramolecular system, with a focus on Pt(iv) prodrugs.

## Introduction

Cisplatin, carboplatin, oxaliplatin are FDA-approved platinum-based anticancer drugs used in clinics worldwide.^1–5^ About 50% of cancer patients receiving chemotherapy are treated with one of these Pt drugs. Their drawbacks are well known and include lack of selectivity, dose-limiting toxic side effects, low bioavailability, and short retention in the bloodstream. The conversion of cisplatin to a Pt(iv) prodrug has been recognized as a promising approach to improve the therapeutic index of Pt-based anticancer agents and reduce side effects.^6^ Pt(iv) prodrugs are generally more inert than their Pt(ii) counterparts, resulting in lower potency and higher toxicity.^7,8^ To achieve their potential as anticancer agents, Pt(iv) prodrugs are reduced intracellularly to the Pt(ii) form.^9–11^ Octahedral Pt(iv) constructs gain two axial ligands upon oxidation of a square-planar Pt(ii) precursor. These extra ligands can coordinate during intracellular reduction. Through modification of these axial ligands, Pt(iv) prodrugs can acquire new chemical and biological properties that facilitate drug delivery or improve therapeutic index.^12–14^ In the past decade, various nanodelivery systems have been investigated for Pt(iv) prodrug delivery, including polymeric nanoparticles,^15,16^ gold nanoparticles,^17,18^ carbon nanotubes,^19–21^ and metal–organic frameworks (MOFs)^22^ among others. Even though a large number of systems have been explored, delivery systems with well-defined size and shape and consistent drug loading are limited. These features are critical for clinical development and are therefore highly sought-after.

Coordination-driven self-assembly represents a well-established methodology for constructing ordered, discrete metal-based nanostructures through controlled aggregation of metals and organic building blocks.^23–27^ These structures have well-defined chemical composition and structural features that can be resolved by NMR spectroscopy, mass spectrometry, and X-ray crystallography. Metal-based polyhedra or cages represent a group of complex inorganic structures accessed through coordination-driven self-assembly.^28^ They usually have a well-defined cavity, which can be used to encapsulate small molecules by host–guest interactions.^29,30^ This property has led to various novel applications in chemistry.^31–33^ For example, a Pd_6_L_4_ cage encapsulates anthracene and phthalimide guests to induce a Diels–Alder reaction with highly unusual regioselectivity.^34^ A Ga_4_L_6_ tetrahedron is reported to be able to stabilize protonated substrates and catalyze the normally acidic hydrolysis of orthoformates in basic solution.^35^ A well-known, pyrophoric species, white phosphorus, can be encapsulated within an Fe_4_L_6_ cage and stably stored in air.^36^

In cancer research, these structures are of interest because their unique chemical and host–guest properties distinguish them from traditional mononuclear metal-based anticancer agents. Exploration of the use of such complexes in cancer research is attracting increasing attention.^37–39^ For instance, a self-assembled Fe_2_L_3_ cylinder provides a novel DNA-binding motif in which the complex inserts at a three-way DNA junction.^40,41^ Recently, a group of Ru-based trigonal prisms has been used to encapsulate small organic anticancer agents for delivery.^42^ This field is still at an early stage, and the role that self-assembled metal complexes will play in cancer research remains to be determined.

**Scheme 1:**
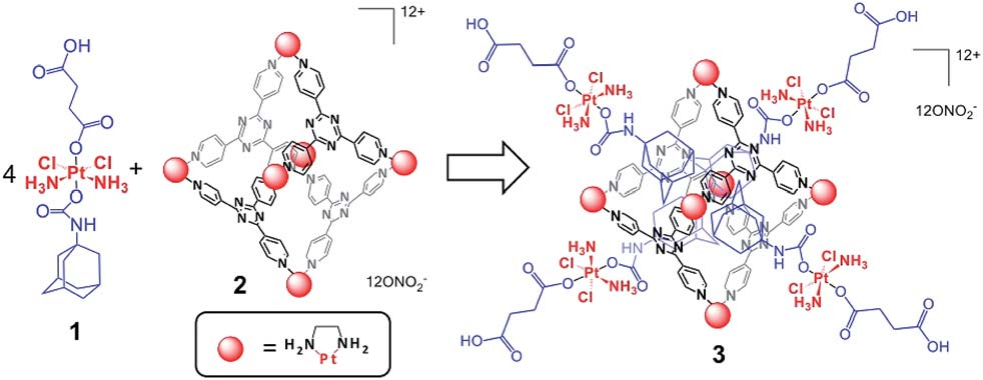
Representation of the host–guest complex (**3**) assembled from the Pt(iv) prodrug (**1**) and the Pt(ii) cage (**2**).

In this article, we describe an innovative design that synergizes coordination-driven self-assembly and Pt-based anticancer drugs. In particular, we developed a novel, well-defined Pt drug delivery system by employing Pt(iv) prodrug technology and coordination-driven self-assembly. This delivery system is composed of cytotoxic Pt(iv) prodrugs and a hexanuclear Pt(ii) cage having low toxicity and high cellular uptake. The cage acts as the delivery vehicle and the Pt(iv) prodrugs as the cargo. As shown in Scheme 1, host–guest interactions between the adamantyl units and the cage drive association of four Pt(iv) building blocks with each cage complex. The high positive charge of the nanoconstruct is proposed to facilitate cellular uptake. Upon entering cells and reacting with biological reductants, the self-assembled supramolecular system is envisaged to release cisplatin and thereby destroy cancer cells.

## Results and discussion

### Pt-based molecular components

The adamantyl Pt(iv) prodrug (**1**) was prepared from cisplatin. Oxidation of cisplatin by H_2_O_2_ forms oxoplatin, *c*,*c*,*t*-[PtCl_2_(NH_3_)_2_(OH)_2_]. Oxoplatin was then allowed to react with 0.9 equiv. of succinic anhydride, resulting in the formation of *c*,*c*,*t*-[PtCl_2_(NH_3_)_2_(OH)(succinate)]. The hydroxyl group of this Pt(iv) compound then performs a nucleophilic attack on 1-adamantyl isocyanate to generate the adamantyl Pt(iv) prodrug **1** in 67% yield.^43,44^ The structure of **1** is supported by multinuclear (^1^H, ^13^C, and ^195^Pt) NMR spectroscopy and ESI mass spectrometry. The ^195^Pt NMR spectrum of **1** exhibited a signal at *δ* = 1250.5 ppm, characteristic of Pt(iv). An isotopically resolved signal at *m*/*z* = 609.9 ([M–H]^–^) was detected in the ESI-MS, confirming the chemical composition. Purity was assessed by elemental analysis (see ESI†).

The hexanuclear Pt(ii) cage, **2**, was prepared according to a previously reported procedure.^45^ In the proton NMR spectrum of **2** in D_2_O, only signals corresponding to the platinum bound pyridyl ligand (*δ* = 8.97 ppm for α-pyridine; *δ* = 8.44 ppm for β-pyridine) and the ethylenediamine moiety (*δ* = 2.72 ppm) were observed. The chemical shifts for **2** are close to the reported values, and their integrations (1 : 1 : 1) agree with the structure. In addition, Diffusion-Ordered Spectroscopy (DOSY) NMR was used to measure the size of the resulting supramolecular complex. The diffusion coefficient (*D*) obtained from DOSY^46^ was used in the Stokes–Einstein equation (*D* = *k*_B_ *T*/6π*ηr*, *k*_B_: Boltzmann constant, *T*: temperature, *η*: dynamic viscosity, *r*: hydrodynamic radius) to afford an estimation of the size of the structure. *D*(**2**) is 1.8 × 10^–10^ m^2^ s^–1^ (Fig. S2†), and the hydrodynamic radius for **2** was determined to be 1.4 nm. This value agrees with the crystal structure of a previously reported Pd analogue.^47^ The cage shows good stability under physiological conditions in the presence of glutathione (Fig. S3†).

### Assembly of the host–guest system

**Fig. 1:**
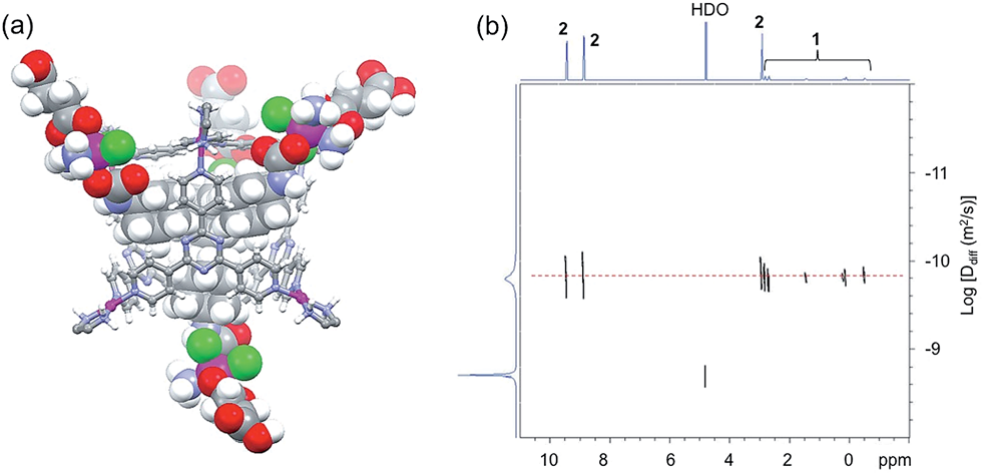
(a) A 3D model of the host–guest complex **3** and (b) DOSY NMR spectrum (400 MHz, R.T.) of **3** (2.8 mM) in D_2_O (*D* = 1.3 × 10^–10^ m^2^ s^–1^).

Formation of the host–guest complex (**3**) was readily achieved by mixing the Pt(iv) prodrug (**1**) and the cage (**2**) in a 4 : 1 ratio at 80 °C with sonication. The overall charge of **3** is 8+ at neutral pH owing to deprotonation of the carboxylic acid groups. Compound **1** has low solubility in water (<500 μM) but becomes readily soluble when mixed with the cage (Fig. S4†). This process is attributed to host–guest interactions between **1** and **2**, with the hydrophobic adamantyl moiety of **1** being encapsulated within the hydrophobic cavity of **2**. 1D and 2D NMR spectroscopy was applied to characterize the formation of the host–guest complex. In the ^1^H NMR spectra (Fig. S5 and S6†), signals from both **1** and **2** undergo significant shifts upon self-assembly. The upfield shift (Δ*δ*) of the adamantyl signals from the Pt(iv) species ranges from –0.71 to –2.36 ppm, whereas downfield shifts of pyridyl signals for the cage were observed around 0.25–0.29 ppm. These significant shifts indicate encapsulation of the adamantyl moiety within the pore and are consistent with the results reported previously with 1-adamantanol.^48^ On the other hand, the NMR signals from the succinate moiety of the Pt(iv) complex display minimal changes in chemical shift (0.01–0.07 ppm), possibly because this ligand extends from the periphery of the cage. As a consequence, the local magnetic environment of the succinate ligand is not significantly altered upon encapsulation. This structural feature was further validated by a 2D NOESY NMR analysis (Fig. S7†). Cross peaks exist between adamantyl signals and pyridyl peaks, but no through-space coupling was observed between the succinate unit and the cage. As shown in Fig. 1a and S8,† a 3D model of **3** generated from Maestro and Spartan reveals these structural features. Additionally, results from a DOSY NMR analysis (Fig. 1b) also support the self-assembly. In D_2_O (*η*_water_ = 8.90 × 10^–4^ Pa s), signals from both the Pt(iv) species and the cage exhibit diffusion coefficients that are almost equivalent, ∼1.3 × 10^–10^ m^2^ s^–1^, whereas **1** alone in DMSO-d_6_ (*η*_DMSO_ = 2.00 × 10^–3^ Pa s) has a diffusion coefficient of 1.6 × 10^–10^ m^2^ s^–1^ (Fig. S1†). Taken together, these results confirm that the Pt(iv) building blocks are encapsulated within the cage. Integrations extracted from the ^1^H NMR spectra indicate their binding ratio to be 4 : 1. The composition of **3** was verified by elemental analysis (see ESI†).

### Release of cisplatin upon reduction

**Fig. 2:**
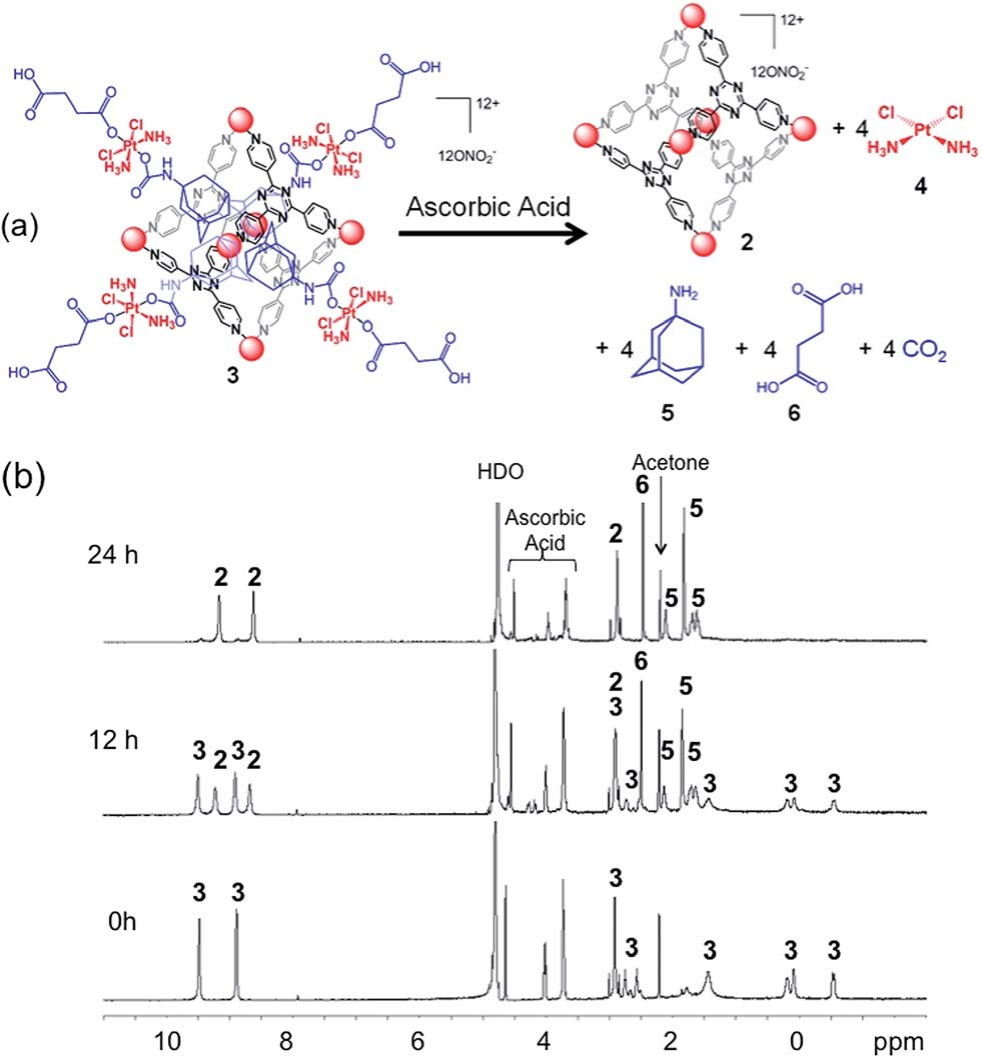
(a) Chemical representation of the reduction of **3** by ascorbic acid and (b) ^1^H NMR (400 MHz, R.T.) spectra showing reduction of **3** (0.8 mM) by ascorbic acid (8 mM) in deuterated PBS over 24 h at 37 °C.

**Fig. 3:**
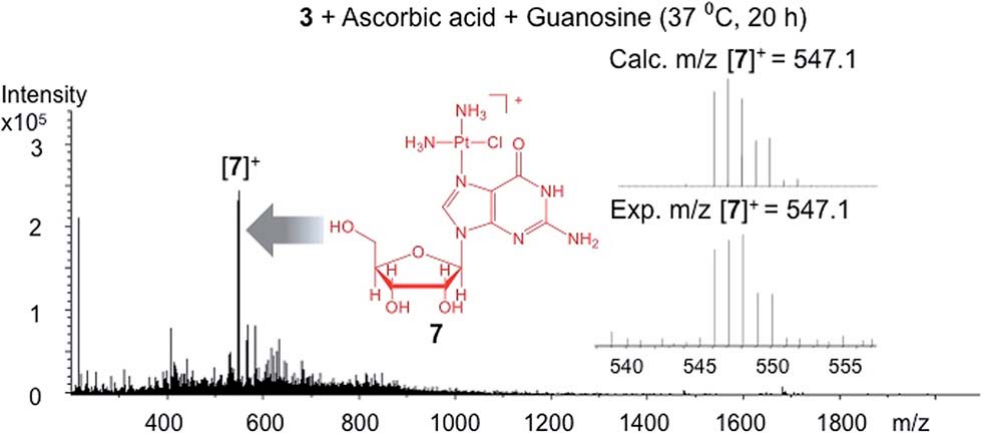
ESI-MS of the reaction mixture of **3** (0.16 mM), ascorbic acid (17 mM), and guanosine (0.62 mM). The cationic adduct **7** forms from the reaction of the released cisplatin and guanosine.

To exhibit anticancer activity, cisplatin must be released from this host–guest system upon reduction. We evaluated the reduction of the system and the release of cisplatin using a variety of methods, including cyclic voltammetry, NMR spectroscopy, and ESI mass spectrometry. The cyclic voltammogram of **3** has three irreversible reduction features (Fig. S9†). The two most cathodic signals have peak potentials of –930 and –810 mV *vs.* Ag/AgCl at a scan rate of 200 mV s^–1^. Electrochemical analysis of the empty cage **2** suggests that these features arise from its triazine core. The remaining signal has a peak potential of –550 mV at an equivalent scan rate, which we assigned to the irreversible reduction of the Pt(iv) prodrug encapsulated within the cage. We also treated **3** (0.8 mM) with 10 equiv. of ascorbic acid (8 mM), a biological reductant, in deuterated phosphate buffered saline (PBS) and monitored the reduction process by ^1^H and DOSY NMR spectroscopy. The reaction is depicted in Fig. 2a. The drug delivery construct is reduced by ascorbic acid and releases cisplatin (**4**), 1-adamantylamine (**5**), and succinic acid (**6**). Upon incubation at 37 °C for 12 h, about 40% of the Pt(iv) species is reduced according to the relative integration of the NMR signals of **1** and **3**. After 24 h, the Pt(iv) compound was fully reduced. Notably, 1-adamantylamine alone does not encapsulate within the cage (**2**) because the amine group is protonated at neutral pH. Disassembly of the supramolecular system was followed by DOSY NMR spectroscopy (Fig. S10†). Upon reduction, the diffusion coefficients of the species giving rise to the succinate and adamantyl signals changed from 1.3 × 10^–10^ m^2^ s^–1^ to 5.0 × 10^–10^ m^2^ s^–1^. In D_2_O solution, the ^1^H NMR signal from the ammine groups of cisplatin was not observed. We used ESI mass spectrometry to confirm that cisplatin was being released. An aqueous solution of **3** (0.16 mM) was incubated with ascorbic acid (17 mM) in the presence of guanosine (0.62 mM) in a total volume of 1 mL. Upon release from **3**, cisplatin is able to covalently bind to guanosine to form the cationic adduct, *cis*-[Pt(NH_3_)_2_Cl(guanosine)]^+^ (**7**). The presence of **7** served as marker for cisplatin release. After 20 h of incubation at 37 °C, ESI-MS of the reaction mixture revealed the formation of **7**, indicative of cisplatin release (Fig. 3, *m*/*z* = 547.1). A control study under identical conditions (20 h, 37 °C) in the absence of ascorbic acid did not yield **7** (Fig. S11†). Collectively, the CV, NMR spectral, and ESI-MS data support the conclusion that cisplatin is released upon reduction.

### Cellular response

**Fig. 4:**
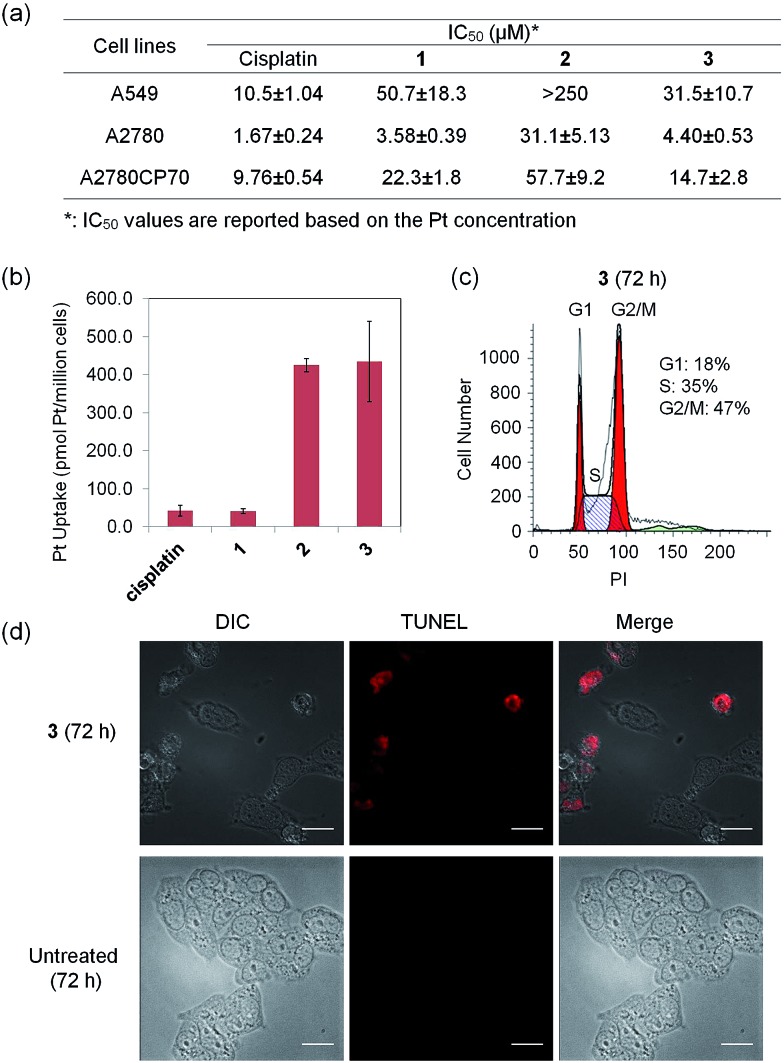
(a) Cytotoxicity profiles of cisplatin and **1–3** against A549 (lung cancer), A2780 (ovarian cancer), and A2780CP70 (ovarian cancer resistant to cisplatin) cell lines, (b) whole cell uptake of the Pt compounds in A2780CP70 cell line ([Pt] = 30 μM, 4 h, 37 °C, 5% CO_2_), (c) flow cytometric analysis of cell cycle of A2780 cells treated with **3** ([Pt] = 30 μM) for 72 h, and (d) cellular images of the TUNEL assay results from A2780 cells treated with **3** ([Pt] = 30 μM) for 72 h (scale bar: 20 μm).

The anticancer activity of **3** was explored in a group of human cancer cell lines. Cytotoxicity profiles of the Pt(iv) prodrug (**1**), the cage (**2**), and the supramolecular system (**3**) were evaluated against A549 (lung carcinoma), A2780 (ovarian carcinoma), and A2780CP70 (ovarian carcinoma resistant to cisplatin) cells. The IC_50_ values (concentration required to reduce viability to 50%) are summarized in Fig. 4a. To permit a valid comparison, all values refer to concentrations of platinum rather than molar concentrations of molecules or complexes. The supramolecular complex **3** displays micromolar potency against all cancer cell lines tested, comparable to that of cisplatin. In A2780CP70 cells, **3** exhibits higher cytotoxicity (IC_50_ = 14.7 ± 2.8 μM) than **1** and **2** (IC_50_ = 22.3 ± 1.8 μM and 57.7 ± 9.2 μM, respectively), which highlights the advantages of the host–guest complex. The elevated cytotoxicity profile of **3** is attributed to high cellular uptake of the cationic cage. We evaluated directly the cell uptake of cisplatin and **1–3** in A2780CP70 cells. The results, shown in Fig. 4b, reveal that **2** and **3** have 10 times greater uptake than either cisplatin or the Pt(iv) prodrug (**1**).

The cytotoxic effect of **3** is attributed to intracellular release of cisplatin, a conclusion supported by observation of effects characteristic of cisplatin on cells. DNA-flow cytometric studies were conducted to identify the incidence of cell cycle arrest upon treatment of cancer cells with **3**. As shown in Fig. 4c and S12,† **3** induces cell cycle arrest in the A2780 cell line. After 72 h incubation, cells arrested at the G2/M phase (40% increase in G2/M population compared to the untreated control). This behavior is consistent with the G2/M cell cycle arrest response evoked by cisplatin.^49^ The host–guest complex **3** is able to kill cancer cells by triggering programmed cell death, or apoptosis. By using a dual staining annexin V/PI flow cytometry assay, the occurrence of apoptosis was investigated in A2780 cells treated with **3**. The results (Fig. S13†) show that **3** can induce apoptosis in A2780 cells after 72 h, similar to the treatment with cisplatin. Compound **3** prompts A2780 cells to undergo early (5.51%) and late (7.42%) stage apoptosis. Apoptotic cells normally exhibit characteristic cellular changes, such as blebbing, chromatin condensation, and DNA and nuclear fragmentation, all of which can be probed by fluorescence microscopy (see Fig. S14† and 4d). Upon incubation of A2780 cells with **3** ([Pt] = 30 μM for 72 h), red fluorescent signals arising from the terminal deoxynucleotidyl transferase dUTP nick end labeling (TUNEL) reaction were apparent, indicative of DNA fragmentation and apoptosis. The evidence compiled from the cell-based experiments described above supports the proposal that **3** releases cisplatin in cells to effect anticancer activity.

## Summary and conclusions

We present a novel approach for developing a delivery system for cisplatin based on the use of Pt(iv) prodrugs and a self-assembled metal-based complex. Pt(iv) building blocks capable of acting as prodrugs that release cisplatin were loaded into a Pt(ii) cage complex *via* host–guest interactions. Upon formation of such a supramolecular system, the cytotoxicity profile of the prodrug was improved because of the high cellular uptake of the cage. Structural features of the system are well supported by NMR spectroscopy, and the biological properties were evaluated by the MTT assay, cellular uptake studies, flow cytometry, and fluorescence microscopy. As a proof of concept, this report described an innovative strategy combining platinum-based medicinal chemistry and coordination-driven self-assembly to produce a well-defined system that delivers cisplatin. The success of this system not only demonstrates the potential for applications of self-assembled metal-based complexes in cancer research, but also provides new concepts for designing novel drug delivery systems.
